# Society Note - ‘How Do We Clean Up the Scientific Record?’

**DOI:** 10.1093/function/zqae028

**Published:** 2024-06-11

This note relates to the following editorial: Alexei Verkhratsky, Ole H Petersen, How Do We Clean Up the Scientific Record?, *Function*, Volume 4, Issue 6, 2023, zqad055, https://doi.org/10.1093/function/zqad055

An independent review has determined that this editorial was not handled independently or peer reviewed in line with American Physiological Society journal guidelines. The opinions stated in this editorial are solely those of the authors.

